# Textile cell-free scaffolds for in situ tissue engineering applications

**DOI:** 10.1007/s10856-015-5656-3

**Published:** 2016-01-22

**Authors:** Dilbar Aibibu, Martin Hild, Michael Wöltje, Chokri Cherif

**Affiliations:** Technische Universität Dresden, Fakultät Maschinenwesen, Institut für Textilmaschinen und Textile Hochleistungswerkstofftechnik, 01062 Dresden, Germany

## Abstract

In this article, the benefits offered by micro-fibrous scaffold architectures fabricated by textile manufacturing techniques are discussed: How can established and novel fiber-processing techniques be exploited in order to generate templates matching the demands of the target cell niche? The problems related to the development of biomaterial fibers (especially from nature-derived materials) ready for textile manufacturing are addressed. Attention is also paid on how biological cues may be incorporated into micro-fibrous scaffold architectures by hybrid manufacturing approaches (e.g. nanofiber or hydrogel functionalization). After a critical review of exemplary recent research works on cell-free fiber based scaffolds for in situ TE, including clinical studies, we conclude that in order to make use of the whole range of favors which may be provided by engineered fibrous scaffold systems, there are four main issues which need to be addressed: (1) Logical combination of manufacturing techniques and materials. (2) Biomaterial fiber development. (3) Adaption of textile manufacturing techniques to the demands of scaffolds for regenerative medicine. (4) Incorporation of biological cues (e.g. stem cell homing factors).

## Introduction

The classical tissue engineering (TE) approach (in vitro expansion of cells seeded on scaffolds and subsequent implantation) has been facing various critical obstacles concerning the translation to the bedside, namely seeding-time, laborious effort and cost [1–3]. Hence, in recent years in situ TE has gained increasing attention [4–8]. In this more straight-forward approach, the body’s own biologic resources and reparative capability are utilized by implanting a cell-free engineered biomaterial (scaffold) into the site of injury, where host stem cells or tissue specific progenitor cells are recruited [2]. In situ TE approaches have been investigated for various possible applications such as vascular grafts [9], nerve [10] and hard tissue regeneration [11].

**Fig. 1:**
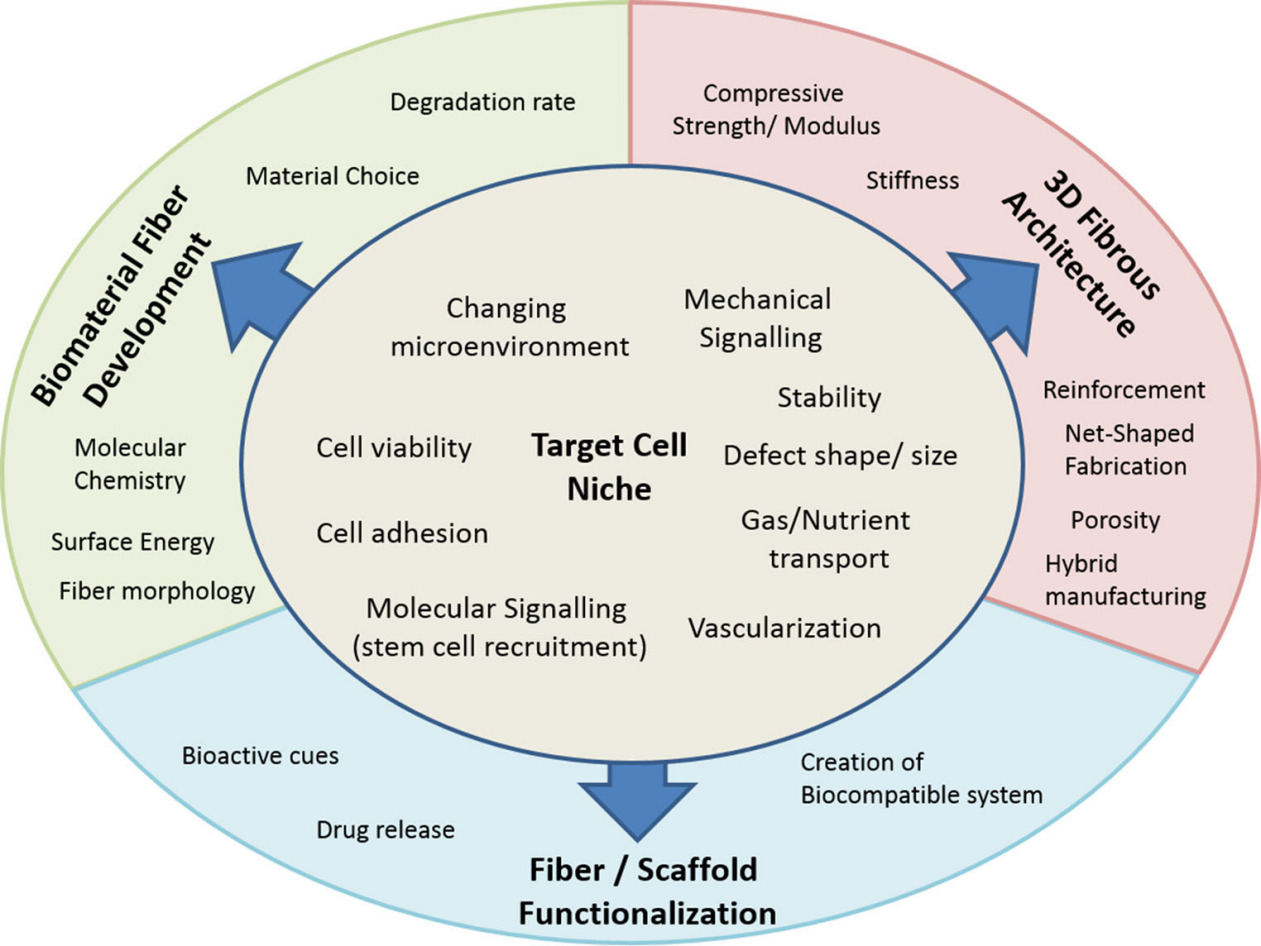
Scaffold development approach based on the requirement of the target cell niche

In recent years the general understanding of the requirements imposed on scaffolds for TE applications has changed towards templates which replicate the target cell niche in terms of their structural architecture and which are capable of adapting to a changing microenvironment, thus providing optimal conditions for tissue-ingrowth, nutrient, gas and biomolecule transport and vascularization [12, 13]. The scaffold architecture should be dictated by the requirements of the target cell niche (Fig. 1). To generate scaffolds with properties tailored to the targeted application, numerous manufacturing methods have been employed. Those comprise solvent casting [14–16], gas foaming [17–19], phase separation [20, 21], emulsion freeze drying [22–24], additive manufacturing (AM) techniques [25–27], electrospinning [28, 29] and other fiber formation techniques [30, 31].

The versatility of textile technology allows for the fabrication of 3D spatial structures with tunable properties in the micro- and macro range [32, 33]. In the past decades, textile manufacturing techniques have been used in a wide range of engineering applications such as fiber reinforced composites [33], construction textiles [34], filtration [35], medical textiles [36] etc. Fiber based techniques including electrospinning have been successfully used for the manufacturing of 3D cell laden scaffolds for classical in vitro and in vivo TE approaches, which are reviewed elsewhere [28, 30]. Through suitable combinations of material, fiber type and manufacturing technique, fiber-based scaffolds can be engineered to obtain properties similar to native tissue and to match critical scaffold criteria [37]. The mechanical properties can be adjusted according to the desired properties of native tissue [38–40]. Besides close structural resemblance of the scaffold to native host tissue, the success of in situ TE approaches strongly relies on the scaffolds capability to recruit host stem cells or tissue specific progenitor cells [2]. Therefore, information-rich scaffolding systems with incorporated “cell homing” or “recruiting” factors are needed [7, 41]. Nanofiber based scaffolds have been reviewed in detail elsewhere [42, 43]. Hence, in this review nanofibers are considered in terms of a major technique for scaffold functionalization and not as structure defining component.

In this article, the benefits offered by micro-fibrous scaffold architectures fabricated by textile manufacturing techniques are discussed:

- How can established and novel fiber-processing techniques be exploited in order to generate templates matching the demands of the target cell niche?
- Which are the problems related to the development of biomaterial fibers ready for textile manufacturing?
- How may biological cues be incorporated into micro-fibrous scaffold architectures?

After a critical review of exemplarily selected recent studies on cell-free fiber based scaffolds for in situ TE, including clinical trials, the findings of this article are concluded in order assess the potential and limitations of cell-free fiber based scaffolds.

## Benefits of engineered fibrous scaffold architectures

**Fig. 2:**
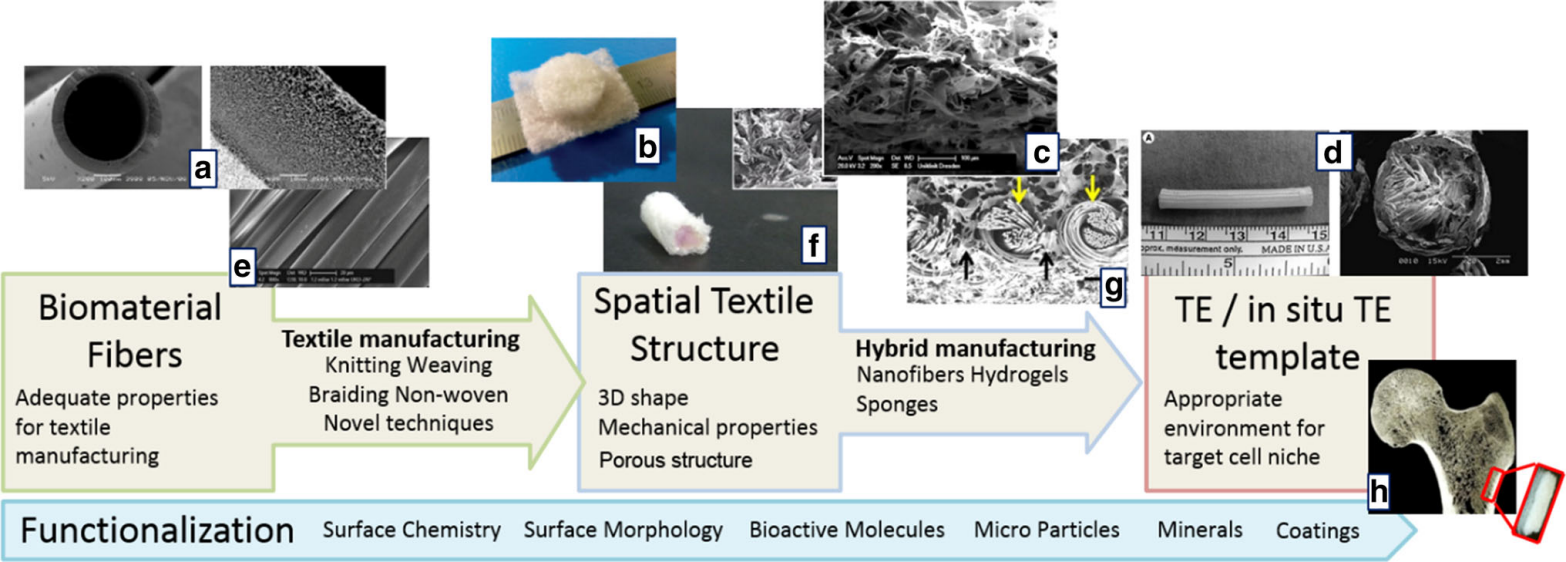
Engineering process of fibrous architectures as templates for TE/in situ TE applications. Image source: **a** [46], **b** [47], **c** [48], **d** [49], **e** [50], **f** [51], **g** courtesy of TU Dresden

Textile technology offers various manufacturing methods to fabricate scaffolds with tailored properties. For centuries, textile manufacturing techniques have been used in the traditional branches of textile industry [44]. Detailed descriptions of the principles of knitting, weaving, braiding and non-woven fabrication as well as their respective characteristics are given elsewhere [32, 33, 45]. This section describes the favors principally given by fiber and textile technology to design scaffolds with adjustable properties matching the target cell niche (Fig. 2). At the same time, limitations and shortcomings of the current state of the art in fibrous scaffold engineering are discussed.

### Textiles: three-dimensional spatial structures with hierarchical porous properties

There are two major groups of fibrous scaffolds for TE and in situ TE applications: Electrospun nanofibrous meshes and microfibrous textiles. Nanofibrous scaffolds have gained increasing attention in the past decade [42]. They are popular due to their high surface- to volume ration, the beneficial scale-dimensions for cell adhesion and the diversity of biomaterials which may be processed [28]. However, nanofibrous scaffolds are restricted to flat, “two-dimensional” pads, tubed membranes or mechanically instable spatial structures in the most cases [42]. The benefit of fibrous engineered architectures which is most often exploited is their reinforcing function [52, 53]. The mechanical properties are identified as a crucial factor for TE scaffolds [49, 54], remarkable coherences between mechanical stability and cell attachment were found [55, 56].

Engineered fibrous constructs are mostly incorporated into scaffolds as either flat textile fabrics or tubular structures made by rolling of flat fabrics or directly produced by braiding or knitting techniques. An issue which is less addressed in studies using engineered fibrous scaffolds is the call for scaffolds with defined pore size and porosity and with 3D shapes which match specific anatomies. To match these requirements, additive manufacturing (AM) techniques have gained increasing attention [25, 57]. The benefits of AM techniques in scaffold manufacturing are underlined in various studies [25, 57]. Besides this, textile manufacturing methods may also be employed for the fabrication of mechanically stable, three-dimensional spatial structures with hierarchical porous properties. With the available conventional manufacturing methods (e.g. flat-knitting, 3D weaving, braiding), net-shaped geometries may be realized [33]. The pore structure of textile fabrics can be adjusted by varying the manufacturing parameters [58]. To learn why there are only little studies using complexly custom-shaped fibrous scaffolds one has to look at the manufacturing methods and the research fields that must be involved.

One main impediment is that industrial textile machines commonly are in scale dimensions that are inappropriate for the fabrication of scaffolds. Furthermore, textile manufacturing of complex 3D-structures requires expert knowledge from textile engineering professionals. There are several examples in which stronger interdisciplinary collaboration could have helped to improve the architecture of the fibrous scaffolds [48, 59–61], thus further improving the positive results. In order to being able to exploit the whole bandwidth of benefits offered by engineered fibrous scaffolds, the technique which suits best for the desired application needs to be identified among the variety of available techniques. For instance, circular knitting could be considered for the fabrication of tubular samples rather than wrapping and suturing knitted flat patches [48, 60]. Manufacturing techniques may also have to be adapted to match the demands of scaffold-fabrication. The trend towards multi-material “hybrid” scaffolds with macro- and nano-scaled elements which most closely mimic the host tissue niche [12, 62] demands flexible manufacturing methods which allow the combination of the fibrous architecture with other materials (e.g. nano-fibers, hydrogels). A manufacturing technique by which fibrous scaffolds are fabricated similar to AM techniques was developed by Hild et al. [47]. Another feature which may be addressed with the help of textile manufacturing methods is the incorporation of nanofibers into 3D stable scaffolds [47, 63, 64]. Such a combination of micro- and nano-scaled elements can be employed to make use of the favorable properties of nanofibers in a mechanically stable 3D spatial environment [26] and to create hierarchical porous structures which are important for scaffold vascularization [65].

### Biomaterial fiber development for use in engineered fibrous scaffolds

**Fig. 3:**
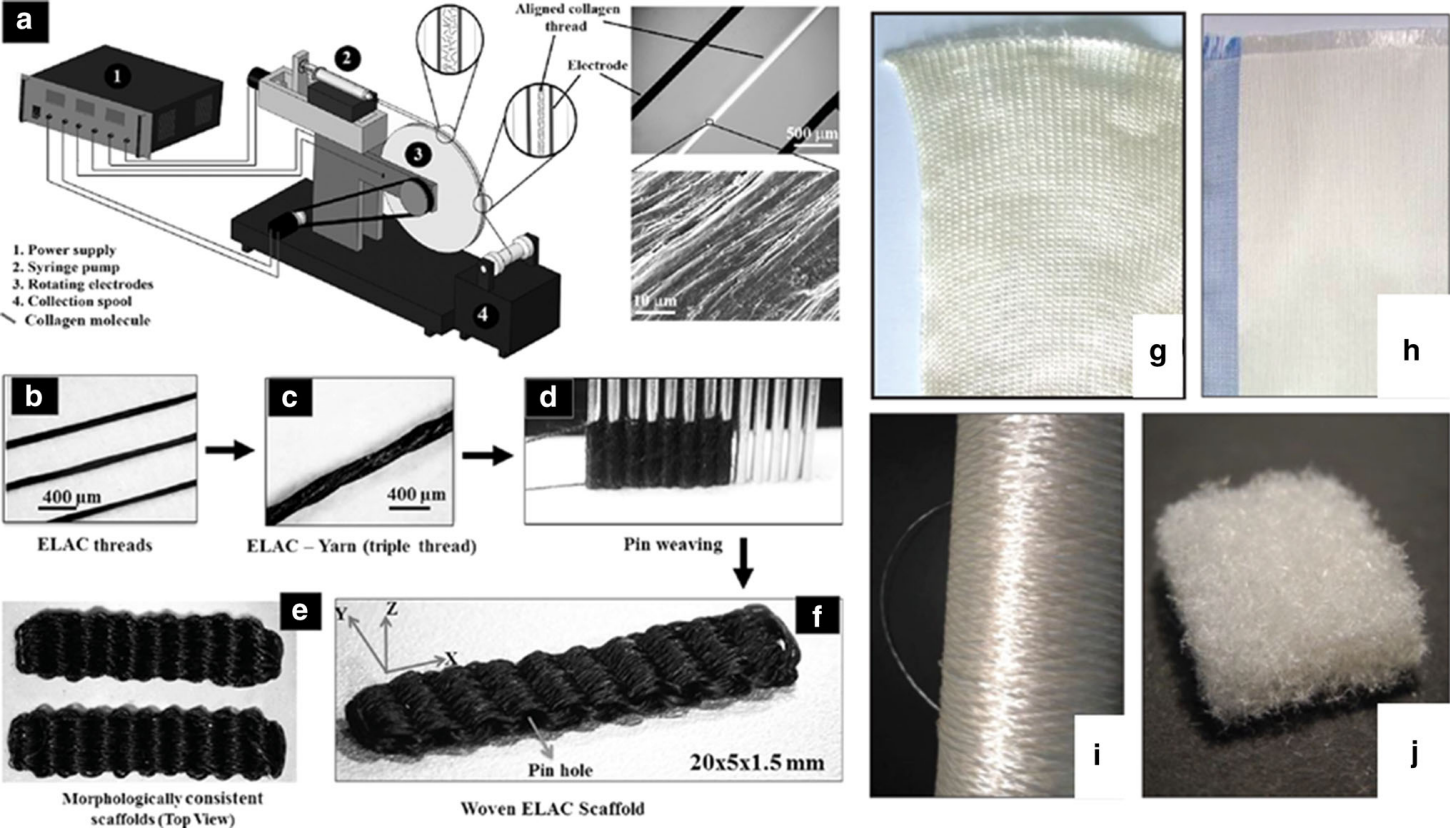
Examples for biomedical fiber development. Schematic of device for the fabrication of endless collagen filament by electrochemical alignment (**a**), electrochemically aligned collagen (ELAC) fibers (**b**), twisted thread (**c**) and pin-woven scaffold made of ELAC fibers (**d**, **e**); Chitosan fibers (**f**), non-woven (**g**), knitted (**h**) and woven (**i**). **a**–**e** from Ref. [80], **f**–**i** from [51]

Theoretically most materials can be processed into fibers by different fiber formation techniques like melt- wet- or dry-spinning [66, 67], electrospinning [68] including nanofiber yarns [69, 70], bio-spinning [71], interfacial complexation [72] and microfluidic techniques [73]. However, fibers which may be used in textile manufacturing processes must meet certain criteria, namely mechanical strength, elasticity, fiber diameter, fiber length, yarn count [45]. To this date, these criteria basically restrict the fiber formation techniques which are suitable for subsequent textile manufacturing methods to melt- wet- and bio-spinning. Due to the above-mentioned reasons, scaffolds made by textile manufacturing techniques are in most cases made of synthetic melt- or wet-spun polymers (e.g. PLA, PGA, PLLA, PLGA, PCL) [74]. To make use of other promising natural or nature-derived biomaterials for the fabrication of stable 3D spatial fibrous scaffolds, intensified fiber development research is necessary. Conventional fiber spinning techniques like wet or melt spinning may generally be used for biomaterial fiber fabrication. However, they often come along with harsh processing conditions (high temperatures, strong/toxic solvents), which could lead to denaturation of the biomaterial during fiber formation or to the presence of toxic substances in the fabricated fibers. For the production of biomaterial fibers, novel approaches have to be followed [75] and existing spinning techniques have to be adapted in order to preserve the microstructure of the biomaterials (i.e. benign solvents, moderate process temperatures). In this section, studies that aim at processing promising biomaterials (collagen, chitosan, regenerated silk, recombinant proteins) into fibers suitable for textile manufacturing techniques are presented (Fig. 3). Detailed descriptions of the respective biomaterial properties can be found elsewhere [76–79].

#### Collagen fibers

Collagen, the major component of native ECM, befits an outstanding role in TE and in situ TE. The beneficial properties of collagen (as explained elsewhere [76]) have been exploited in numerous studies in all sub-branches of TE [81, 82]. Since the first study on reconstituted collagen fibers by extrusion from acidified collagen gels different routes have been followed in order to develop aligned fibers which possess sufficient mechanical strength for their intended use and replicate the micro and nano-structural composition of native collagen [83]. In recent years, significant progress towards these goals has been made using extrusion and electrochemical alignment techniques, respectively.

Wet-spinning of collagen fibers can be realized by extruding acidified collagen gels into a pH-neutral buffer, where in vitro fibrillogenesis takes place [84]. Based on this approach, research has been conducted in order to improve the mechanical strength [85], increase production rates [86], adjust the fiber properties by using different co-agents, solution concentrations and collagen sources [87–89] and examine fiber crosslinking [90]. Fiber-diameters of 10–2000 µm can be realized [83]. Although D-banded collagen fibers can be verified [91], it remains unclear whether native-type collagen without substantial proportions of gelatin (denaturalized collagen) can be produced by extruding acidified collagen [83]. Furthermore, the usage of polyethylene glycol (PEG) in the fiber formation buffer increases the wet fiber strength but results in non-pure collagen fibers [92]. In addition, extruded collagen-fibers are mechanically weak. Crosslinking may elevate the ultimate strength of to an adequate level (10.5 MPa), but at the same time negatively influences the cell-seeding properties [93–96]. Another challenge lies in the fabrication of multi-filament yarns. While most studies describe the fabrication of single fibers, there is little and unsatisfying work on the production of multi-filament collagen yarns that may be used for textile manufacturing [97]. The limitations described above render extruded collagen fibers impractical for textile manufacturing methods.

By describing an electrochemical alignment technique, Akkus et al. published pioneer work on engineered collagen fibers. The principle of axial alignment of dialyzed collagen along the isoelectric point and subsequent fibrillogenesis in phosphate buffered saline (PBS) at 37 °C and genipin crosslinking was first described by Cheng et al. [75]. By adjusting the genipin-crosslinking protocol, the mechanical strength of the electronically aligned collagen (ELAC) could be elevated to 109 MPa, which is in the range of native tendon [98]. Younesi et al. developed a rotating electrode device for the continuous fabrication of ELAC fibers (Fig. 3a–e) [80]. However, the production speed remains unclear. They twisted three single fibers (ultimate tensile strength 20–50 MPa, 0.1–0.15 mm fiber diameter) to a collagen thread (ultimate tensile strength 65 MPa) and used a hand weaving technique to produce a 3D bio-textile made of pure collagen fibers. hMSC’s proliferated and distributed throughout the highly porous scaffold underwent tenogenic differentiation even though no growth factors were added [80]. However, the authors state that thinner filaments could be beneficial for cell alignment. Thinner filaments would be also useful for textile fabrication processes. If in vivo examinations of this novel collagen-fiber material prove successful, electrochemical alignment could be a breakthrough technique for the fabrication of pure collagen fibers.

Besides the promising developments in collagen fiber development, there are some unsolved problems. For example, there is a controversy about the correct cross-linking procedure or whether collagen should be cross-linked at all [99]. Furthermore, current studies describe collagen fiber production on the laboratory scale in which single fibers or fiber bundles comprising up to six fibers are produced [94]. From the materials’ engineering point of view, the next steps should include up-scaling of the fiber fabrication process with the goal to produce multifilament yarns and processing the collagen yarns on textile machinery. Another critical issue remains the verification of the ultrastructural features of collagen in fibers labeled as “collagen fiber”. There are studies on “collagen” fiber scaffolds that lack the verification of collagen and in which the source of the collagen fibers remains unclear [100, 101].

#### Chitosan fibers

Due to its favorable properties (e.g. abundant availability, osteoactivity, promotes wound healing, anti-bacterial effects) Chitosan (CS), a partially deacetylated derivative of Chitin, has been widely used in applications for regenerative medicine [102, 103]. Chitosan fibers may be produced by electrospinning [104], microfluidic spinning [105] or wet spinning techniques [51, 106–108].

Li and colleagues reported the fabrication of CS fibers from glycine chloride ([Gly]Cl) ionic liquid spinning dopes. 5–7 wt%. CS dissolved in in 4 wt% aqueous acetic or [Gly]Cl solutions was spun with a spinneret (20 holes, 80 µm Ø). The filaments were coagulated in a dilute Na2SO4/C2H5OH bath. Adequate mechanical properties (maximum breaking tenacity: 3.77cN/dtex, maximum initial modulus: 2.3 cN/dtex) were registered [109]. Yan an co-workers used chitin nanocrystal (ChiNC) as nanofiller to reinforce CS fibers spun according to 109, leading to increased mechanical properties [110]. Chitosan microfibers reinforced by chitin nanofibrils were studied by Yudin et al. [111]. The incorporation of 0.1–0.3 wt% of chitin nanofibrils into chitosan matrix led to an increase in strength and Young modulus.

Toskas and colleagues successfully developed an industrial-scale process to generate pure chitosan multifibers (Fig. 3f–i) [51]. They dissolved up to 8.5 wt%. CS in aqueous acetic solutions. The CS solution was pressed through a spinneret (150–600 holes, 20–120 μm Ø) and led through a coagulation bath composed of NaOH/EtOH. The fiber diameter could be adjusted from 20.7 to 36.0 µm. Tensile strength of 15.9 cN/tex was achieved. Woven and knitted fabrics with adequate mechanical properties could be manufactured [51]. Textile scaffolds made of these CS fibers were tested in vitro, yielding promising results for further use in TE applications [47, 112]. Chitosan-based hyaluronic acid fibers were developed by Yamane et al [113]. They used spinning dopes of 3.5 % CS in 2 % aqueous acetic solutions, a spinneret with 50 holes and 0.1 mm Ø and a calcium coagulant bath. Hyaluronic acid (HA) was added in an aqueous methanol solution coagulation bath. CS-HA fibers showed higher tensile strengths (168.2 ± 7.0 N/mm^2^) than CS only fibers (87.4 ± 2.0 N/mm^2^) [113]. This fiber-type was used for the fabrication of 3D woven scaffolds [114]. Implantation into cartilage defects in rabbits led to regeneration of hyaline-like cartilage [108, 115].

Similar to collagen fibers, the successful works on chitosan fiber development call for intensified studies about their in vivo behavior in order to utilizing fibrous chitosan structures as scaffolds for TE and in situ TE applications.

#### Regenerated silk fibers

Silk fibroin-based scaffolds have been used in various TE and in situ TE applications [116]. Although silk fibers directly harvested from the spinning gland of spiders (Ampullate/Dragline fibroins) or silkworms (Bombyx mori) provide excellent properties (e.g. mechanical stability, beneficial cellular reactions, biodegradability) they also have shortcomings in terms of material inhomogeneity, varying material properties and the low availability of spider silk [79, 117]. To elude these problems which come along with the use of biospun fibers, extensive research has been put on the development of wet-spinning techniques for regenerated silk fibers.

The fabrication of regenerated silk fibroin (RSF) fibers is a challenging subject. Critical factors are the molecular weight and concentration of silk fibroin [118], the solvent system [119, 120], the solidification rate of the spinning dope [121], the post-drawing ration [122] and the preservation of flexibility in the dry state [123]. Conductive RSF may be developed by the incorporation of multiwalled carbon nanotubes (MWNTs) [124]. Their biocompatibility may be improved by adding calcium chloride to the spinning solution [125]. Due to the limited availability from natural sources, recombinant production is especially attractive for spider silk proteins [117]. Synthetic recombinant *Major Ampullate* spider silk fibers were produced by self-assembly [126–128] or wet-spinning [129–131]. There are studies about the recombinant production and subsequent fiber-spinning of other proteins than silk fibroins, e.g. silk-elastin like proteins [132], Amyloid Protein [133], honeybee silk [134] or Keratin [135]. Those materials could be used to create novel fibers with favorable properties for their use in regenerative medicine. However, these attempts had limited success regarding the mechanical properties of the fibers.

### Fiber and scaffold functionalization for the incorporation of stem cell homing factors

Fiber science provides useful techniques for the incorporation of functional substances into scaffolds. Much research has been conducted on drug-loading and release of fibers [136–138] and fiber based scaffolds [139]. The use of hollow fibers with interconnected micro-pores through the fiber wall has been studied for TE applications [140]. Fiber surfaces can be functionalized with a diversity of nano- and micro-particles in order to improve certain scaffold properties [141, 142]. The available techniques for fiber functionalization and drug-release could also be used for the incorporation and sustained release of biological cues such as stem cell homing factors into fibrous architectures. The possible mechanisms for the loading of fibers with biological cues are discussed in detail elsewhere [62].

Despite the manifold possibilities to functionalize fibers, the only aqueous coatings [143–145] and gels [48, 63, 146] have been used for the incorporation of biological cues into textile scaffolds. Effective stem-cell recruitment by coating a technique was achieved by Erggelet et al. [144]. They prepared cell-free scaffolds by cutting commercially available non-wovens of polyglycolic acid (PGA) into the desired shape and soaking them with hyaluronic acid. Directly prior to implantation into full-thickness articulate cartilage defects of merino sheep, the scaffolds were soaked in autologous sheep serum which served as chemo-attractant. Three months after implantation, the formation of a cell-rich repair tissue of cartilaginous appearance was observed. The authors conclude that the scaffold allows the in situ recruitment of mesenchymal stem cells (MSCs) by serum as a chemo-attractant and subsequent guidance of the progenitor cells towards formation of cartilage repair tissue [144]. A similar scaffold system (PGA non-woven soaked with hyaluronic acid and allogenic serum) was used for the regeneration of the intervertebral disc in rabbits [145]. In another study, coating of knitted polyester vascular grafts with fibronectin (FN) and the stem cell homing factor SDF-1 alpha led to positive results [143]. As these findings suggest, coating is a rather simple technique that can easily be used directly prior to implantation, but it comes along with the limitation that the biological cue is only present on the fiber-surface, which impedes time-dependent release kinematics.

**Fig. 4:**
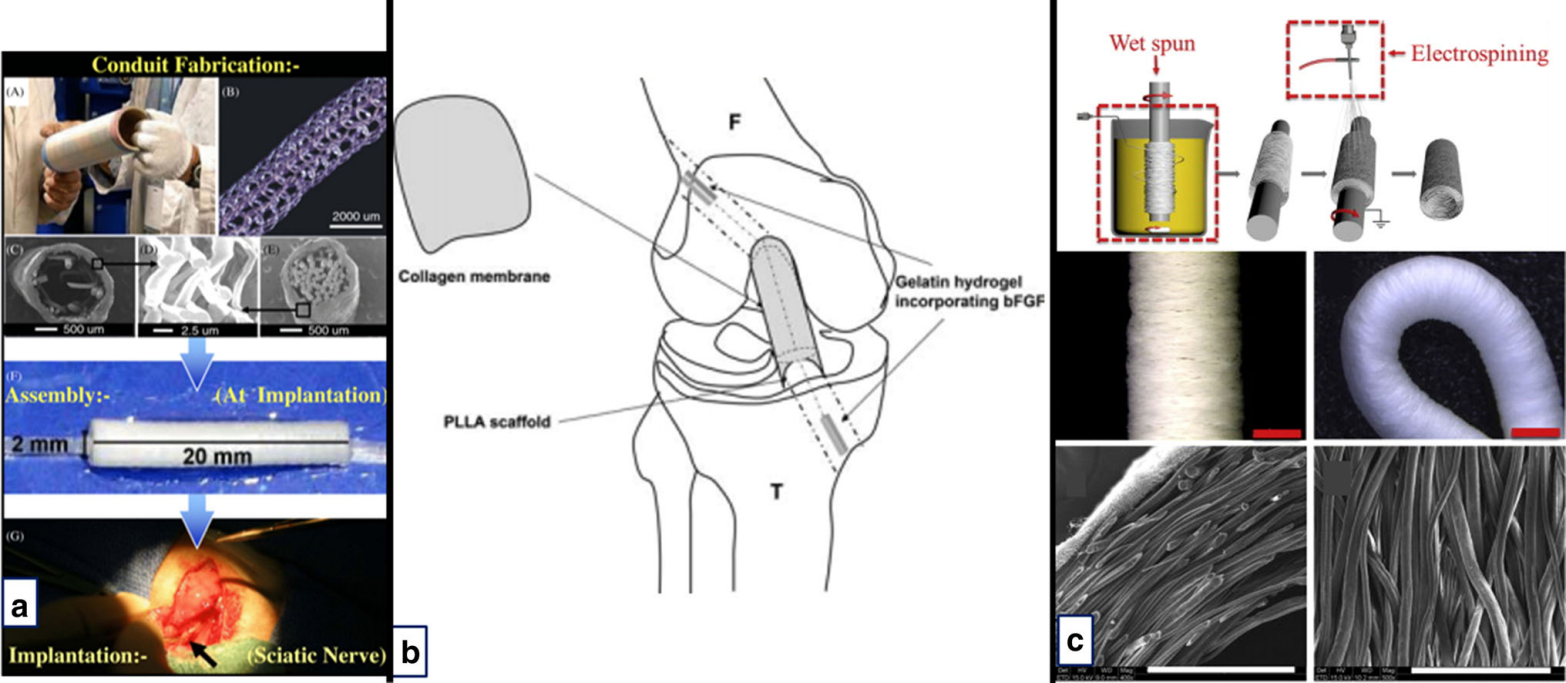
**a** Engineering process of multimodal nerve conduit based on a tubular knit coated with electrospun nanofibers and incorporated with aligned guidance fibers [63]. **b** Scaffold design based on the requirements of different implantation sites: Hydrogels incorporating bFGF localized to the bone tunnels, collagen wrapping in the joint cavity [146]. **c** Hybrid vascular scaffold made of wet-spun microfibers and electrospun nanofibers; manufacturing scheme, photographs and SEM images of scaffolds [64, 147]

Hydrogels may be used for local release of biological cues. For the regeneration of anterior cruciate ligament (ACL) defects in rabbits, Kimura et al. incorporated braided poly-L-lactic acid (PLLA) scaffolds with a basic fibroblast growth factor (bFGF) loaded hydrogel in the region of bone and with a collagen wrapping in the joint cavity [146]. The authors observed significant bone regeneration around the scaffold in the bone tunnel, which could have been supported by enhanced cell migration due to local bFGF release (Fig. 4b) [146]. Shen and co-workers developed a bioactive scaffold made of knitted silk and a collagen sponge with incorporated cell homing factor SDF-1 alpha [48]. This scaffold for Achilles tendon regeneration led to a reduction of inflammatory cells, SDF-1 alpha caused increased selective recruitment of fibroblast-like cells. Four weeks post-surgery, enhanced local endogenous SDF-1 alpha and extracellular matrix (ECM) production was registered [48].

The described findings show that the incorporation of biological cues into textile scaffolds for in situ TE can lead to favorable outcomes. With the help of available fiber functionalization techniques, a broader range of substances could be incorporated and programmable release kinetics of biological cues from degradable fibers could be realized.

## Textile cell-free scaffolds used for in situ tissue engineering

This section gives an overview on recent studies in which fibrous scaffolds are used for in situ TE applications. A focus is put on the textile substrates and their incorporation into the scaffold system. Only the most important features of the different manufacturing techniques are discussed in order to point out their benefits for their use as cell-free scaffolds.

### Knitted scaffolds

With knitting technology, three-dimensionally net-shaped geometries are easily realizable [148–150]. Due to their highly ordered loop-structure [151], knitted fabrics are generally more elastic than woven or braided structures. Using different knitting techniques and patterns (e.g. jersey, interlock), the mechanical [152] and porous [153] properties of knitted scaffolds may be tailored to suit the specific demands of most sub-areas of regenerative medicine [37]. This section describes successful studies using knitted cell-free scaffolds, undermining the versatility and suitability of knitting technology for the fabrication of cell-free scaffolds.

A scaffold design based on the requirements of the target cell niche, by logically combining different manufacturing techniques and materials, was realized by Quigley et al. [63]. They knitted a sheath from PLA monofilaments (90–100 µm Ø) which was coated with electrospun PLA nanofibers to obtain controlled pore sizes favorable for nerve conduits <2 µm [154]. This sheath was filled with two types of aligned (PLGA) guidance fibers (30–40 µm Ø) which are supported by an alginate hydrogel impregnated with neurotrophic factors (NT-3 or BDNF with LIF, SMDF and MGF-1) to provide neuroprotection, stimulation of axonal growth and Schwann cell migration (Fig. 4a). After implantation into 12 mm sciatic nerve defect in rats for 4 weeks, patent regenerated nerves were observed, in which axons grew through and beyond the conduit. It was found that the aligned PLGA fibers support guided Schwann cell migration and neuron outgrowth [63]. This study shows that intelligent scaffold design that combines different manufacturing techniques, together with the incorporation of biological factors, can lead to favorable results. However, with the availability of more appropriate (biomaterial) fibers, the outcomes could be further improved.

The elastic structure of knits is useful where structures that have to adapt to size changes are required. Matsumara et al. implanted scaffolds made of PGA knitted fibers + l-lactide/ε-caprolactone (PCL) copolymer sponge with outer PGA and PCL copolymer monofilament reinforcement into the inferior vena cava/left pulmonary artery of beagle dogs for potential application in pediatric cardiovascular surgery [155]. The initial strength of 9.3 N per 1 cm width was lost by 1 month; the scaffold was degraded by hydrolysis within 6 months, being replaced by native tissue. The scaffolds adapted their shapes after implantation, which is an important feature for its targeted use in pediatric surgery [155, 156]. The authors also point out the cost-effective and time-saving procedure compared to a cell-based approach using the same biodegradable scaffold system which was already tested in clinical trials [157, 158]. However, the scaffold design was not optimized for the proliferation of vascular smooth muscle cells.

Mechanical stability and elasticity are among the most important scaffold-features for skin regeneration [54]. The use of fibrous structures enables the promotion of angiogenesis by mechanical stimulation in vivo [159]. In a cell-free approach, a warp knitted PLGA multifiber mesh (PLGAm) (25 filaments/yarn; 15 µm filament Ø) was used to improve the mechanical stability of porous collagen-chitosan sponges (CCS) [49]. Wet-state tensile strengths of 2.79 MPa could be achieved [159]. After implantation in full-thickness skin defects of rats, the PLGAm/CCS scaffolds inhibited wound contraction, effectively promoted cell infiltration, neotissue formation and blood vessel ingrowth. 8 weeks post-surgery, the tensile strength of the repaired skin reached up to 73 % of normal skin [49].

### Woven scaffolds

Compared to knits, woven fabrics may be designed to achieve higher tensile strength and less elasticity. Besides flat “2D” fabrics, 3D weaving technology allows for the generation of defined spatial geometries [160], which are widely used in fiber reinforced composite applications [33] and also in TE applications [39, 161–163]. With 3D weaving, the through-plane strength of woven fabrics can be improved considerably [32].

Most of the times, weaving techniques are used when high mechanical properties are required. Therefore, popular fields of applications are vascular grafts [147, 164], tendon regeneration [165] or hernia repair [59].

Yokota an colleagues designed the mechanical properties of a small-caliber vascular graft (4 mm Ø) made of a type I bovine collagen microsponge compound with a cylindrically woven biodegradable and absorbable polymer tube (airspun core (PLA)-sheath (PGA) compound yarn), so that initial tensile modulus and tensile strength of the woven grafts (tensile strength: ~30 MPa, modulus: ~200 MPa) were much higher than of native carotid arteries (tensile strength: ~8 MPa, modulus: ~60 MPa) [164]. With absorption of the PGA fibers, tensile strength and modulus decreased to a level that was almost equivalent to that of the native carotid artery of mongrel dogs. An endothelial cell monolayer, smooth muscle cells and a reconstructed vessel wall with elastin and collagen fibers could be observed 12 months post-surgery [147, 164]. In another study concentrating on the mechanical properties, Derwin et al. used a commercially available woven PLA sheet (X-Repair) for the augmentation of rotator cuff defects in dogs [166]. They observed that directly after implantation the repair augmentation significantly increased the ultimate load by 23 % compared to un-augmented repair, while the stiffness remained unaltered. At 12 weeks, the PLA scaffold augmented repairs demonstrated significantly less tendon retraction and significantly greater cross-sectional area (137 %), stiffness (26 %), and ultimate load (35 %) than did repairs that had not been augmented [166]. The same material was also positively evaluated in an initial-load test of augmented human cadaver rotator cuffs [167].

Inui and co-workers studied different surface qualities of a cell free scaffold in a rabbit rotator cuff defect model [165]. They fabricated a woven fabric made of PLLA fibers with a smooth surface on the outside and a rough (pile-finished) surface on the inside [168]. The scaffolds were implanted into defects of the infraspinatus tendon. The rough surface allowed better cell migration while the smooth surface prevented cell attachment. No significant difference in ultimate failure load between scaffolds and the control group (reattached natural infraspinatus tendons) could be observed. 8 weeks post-surgery, the failure strength of intact infraspinatus tendons was regained [165]. This study shows that adjusting surface structures on the macroscopic level may be used to control cell adhesion and migration. The versatility of weaving technology to create 3D structures and fabrics with defined porous properties remain largely unexploited in studies using woven cell free scaffolds.

A cell-free scaffold system made of hand-woven meshes from native dragline silk of the spider *Nephila spp.* for appliance in hernia repair was developed by Schaefer-Nolte et al. [59]. To investigate their usage as fascia replacement material, the meshes and two compare groups (commercially available hernia meshes Ultrapro and Surgisis) were tied onto the paravertebral fascia of rats. The relative elongation of the spider-silk meshes was higher than the relative elongation of Ultrapro and Surgisis, demonstrating the spider-silks’ superior adaptability to changing body shapes. Rapid cell migration into the spider silk meshes and milder immune responses than in the compare croup were registered. Complete degradation was observed after 4 months. However, the authors point out that the functionality of spider silk meshes has to be validated in real hernia models [59]. This study points out the usefulness of novel biomaterial fiber materials. At the same time, it becomes obvious that intensified research is necessary before such materials can be processed with textile manufacturing methods.

### Braided scaffolds

Braiding generally leads to rope-like structures, either dense, hollow, or with embedded core fibers. More complex braiding techniques allow for the fabrication of 3D net-shaped structures [169]. Due to the fiber orientation in a specific angle to the braiding direction and the resulting mechanical properties of braided structures, braiding is among the most common scaffold fabrication techniques used in ligament TE [170].

Aurora et al. investigated PLLA/PGA braid reinforcement as a method for engineering the mechanical properties of lyophilized human fascia patches [171]. The authors conclude that the patch reinforcement may be sufficient to provide mechanical augmentation to rotator cuff repairs and minimize tendon retraction [171]. The same scaffold model (PLLA braid reinforced fascia) was used for rotator cuff repair in dogs [172]. While at time zero the ultimate load of the augmented repairs was 46 ± 25 % more than non-augmented repairs. However, the ultimate load did not increase with time [172]. Hence, the mechanical strength lost by scaffold degradation could not be compensated for by regenerated tissue. In another study, Cooper et al. used multifilament PLLA fibers fabricated into 3D square braids for ACL regeneration in rabbits [173]. Scaffolds seeded with ACL cells were compared to cell-free scaffolds. The initial tensile properties of the braided scaffolds were similar to that of native tissue. Poor tissue ingrowth was observed 4 weeks post-surgery. After 12 weeks, only the cell-seeded scaffolds showed excellent tissue infiltration and revascularization. The cell-seeded scaffolds also retained more of their tensile strength than the cell-free scaffolds [173].

The problems identified in the above-described studies may occur since the scaffolds were not consequently designed based on the requirements of the host tissue. The sole use of cell-free scaffolds without the incorporation of biological cues seems insufficient for ligament regeneration.

In the subject of nerve regeneration thorough research has been conducted on braided, collagen coated PGA tubes with incorporated collagen sponges or fibers. This scaffold type was successfully applied for the regeneration of various different types of nerves in animal models [174–178] prior to its use in clinical studies [179–181] (see “Clinical Studies”). The same workgroup also developed a nerve guide tube based on the well-established PGA-collagen scaffold for the repair of long nerve defects [50]. They used braided scaffolds of PLLA monofilaments and PGA multifilament yarns (1:1) with a collagen coating for the repair of long nerve defects in beagle dogs. Compared to scaffolds consisting of PGA-collagen only, the PLLA-PGA-collagen scaffolds led to faster functional recovery. The lumen structure which is necessary for nerve regeneration was maintained for 12 months. The tubular scaffolds also allowed for the development of normal tibialis anterior (TA) muscle cells [50].

### Non-woven scaffolds

Since their pore size is accurately adjustable, non-woven fabrics are commonly used for filtration applications [35]. This beneficial property has also been exploited in TE applications, especially for cartilage engineering [182–185] and biomaterial evaluation [186–189]. As well as in classical TE approaches, along with the use of collagen-matrixes [190–192], non-woven fabrics are desirable materials for in situ cartilage regeneration.

Yokoya and co-workers developed a cell-free tendon-bone insertion made of a PGA nonwoven sheet for the repair of full-thickness rotator cuff defects in rabbits [193]. Compared to a PCL scaffold used in a control group, the defect sites showed a well-arranged fibrocartilage layer, mainly regenerated by type III collagen. Mechanical examinations revealed significantly higher values in tensile strength [2.36 MPa (PGA) vs. 1.80 MPa (PCL) after 16 weeks] and Young’s modulus [5.53 MPa (PGA) vs. 3.74 MPa (PCL) after 16 weeks] [193]. Another scaffold system for rotator cuff regeneration consists of sheets of non-woven chitin fibers implanted into defects of the infraspinatus tendon [194]. Compared to untreated defects, the scaffolds increased cell numbers and improved collagen fiber alignment. However, the scaffolds possessed inadequate mechanical strength [194]. With a molding technique, Yang et al. fabricated non-woven nerve-guidance conduits made of *bombyx mori* silk fibroin oriented fibers cast in fibroin solution [195]. The grafts were used to bridge a 10 mm gap in sciatic nerves in rats. The scaffolds showed favorable maximum fracture strength (5.5 MPa) and compressive strength (2.5 MPa) in the wet state. They could promote peripheral nerve regeneration. The authors suggest that introducing neurothropic factors could further improve the scaffold, thus becoming a real alternative for nerve autografts [195]. From the described studies, it becomes clear that researchers do not make adequate use of the possibility to adjust the porous properties of non-wovens. Designing the nonwovens based upon the relations of porosity and cell-material interaction should be considered in order to exploit the benefits given by non-wovens.

Zhu and colleagues pursued a design-approach for vascular grafts which allows vascular smooth muscle cells (VSMCs) infiltration and their circumferential alignment (Fig. 4c) [64]. Therefore, they fabricated a bi-layered scaffolds composed of wet-spun circular aligned PCL-fibers covered with an electrospun PCL coating. They obtained vascular grafts with mechanical properties similar to native arteries of rats. The scaffold design plays a key role for the successful cellular integration: The large interconnected pores enabled the VSMCs to infiltrate the graft wall and the circumferentially aligned fibers served as topographical guidance [64].

### Clinical studies using cell-free fiber based scaffolds

Similar to in vitro TE, there are comparatively few in situ TE approaches which have been evaluated in clinical studies. The demand on in situ TE as an approach that avoids the major disadvantages of classical in vitro TE and thus may be transferred to clinical application in a more straight forward way needs to be manifested in clinical case studies. Especially for nerve regeneration, cell-free fiber based scaffolds were successfully used in clinical case studies. The easy fabrication of tubes by textile manufacturing techniques and their possible off-the-shelve availability support their use as a cost-effective treatment of nerve defects. Vascular TE also benefits from fibrous tubes; however there are few clinical studies on in situ vascular TE [9]. This section describes promising clinical case studies using cell-free fiber based scaffolds.

Remarkable clinical application studies in the field of nerve regeneration were conducted by Inada et al. [181]. They developed a braided, collagen coated PGA tube filled with a collagen sponge (70–80 % collagen type I, 20–30 % collagen type III) for the regeneration of large peripheral motor/sensory nerve defects and for causalgia-treatment, respectively. The grafts had a PGA wall thickness of 50 µm and tube-diameters of 4 mm and were used to bridge nerve gaps of up to 65 mm in length [174]. After implantation, the patients experienced functional recovery of their finger/foot [179], regained the voluntary ability to lift their eyebrow [181] and regained full use of their fingers after causalgia and allodynia had disappeared [180]. The authors conclude that their PGA/collagen scaffold is a promising option and viable alternative for conventional nerve grafts. In another clinical study on nerve-repair, Aberg and co-workers used non-wovens made of resorbable poly[(R)-3-hydroxybutyrate] (PHB) fibers for the treatment of complete median and/or ulnar nerve injuries at the wrist/forearm level by wrapping the PHB non-woven pads around the nerve ends in a tube-like fashion [196]. Compared to standard end-to-end suturing, the treatment with PHB non-wovens may be advantageous regarding sensory recovery and manual muscle tests. The authors point out, that the wrap-around technique also offers practical advantages to the surgeon [196]. Currently, with the Neurotube^TM^ conduit (Synovis Life Technologies, St. Paul, USA) there is one commercially available and FDA approved nerve graft made of woven PGA fibers [197–199].

Fibrous cell-free scaffolds for in situ reconstruction of small joints were tested in clinical studies by Honkanen, Tiihonen et al. The scaffold system made of knitted (P(L/D)LA) fibers which was investigated by Waris et al. in a pig model [200] was used for the reconstruction of metacarpophalangeal joints in rheumatoid arthritis patients [201–203]. The knitted interposition scaffold was compared to Swanson silicone implants. After 2 years the improvement in clinical assessments was comparable in both groups, except for better maintenance of palmar alignment in the Swanson group. The lack of implant fractures and intramedullary osteolysis were advantages of the knitted implant [202]. After a mean follow-up of seven years, satisfactory pain relief was registered, but the function was limited. The patient satisfaction was similar in the silicone implant group and in the knitted implant group. The authors conclude that the main clinically relevant outcomes are similar and that due to soft tissue deficiencies long-term function and alignment problems cannot be resolved with PLDLA interposition implant [203]. In reconstruction of the destructed trapeziometacarpal joint in arthritic patients the P(L/D)LA) knitted scaffold was compared to tendon interposition [204]. The authors conclude that bioreplaceable interposition arthroplasty using the knitted scaffold works at least as well as tendon interposition and that the operation using the knitted scaffold is easier [204]

**Table:** 

Targeted application	Materials/manufacturing techniques	Function of fibrous structure	Biological cues/clinical application	References
Knitting				
Vascular grafts	PGA knitted fibers + l-lactide/PCL copolymer sponge with PGA/PCL copolymer monofilament reinforcement	Initial reinforcement; Elasticity for shape adaption	–	[155, 156]
	Knitted polyester; commercially available vascular grafts	Mechanical stability	Fibronectin SDF-1 alpha	[143]
	Knitted PGA/collagen microsponge + woven PLLA (outer layer)	Outer layer: reinforcement; inner layer: porous environment promoting in situ cellularization	–	[205, 206]
	Knitted PLGA (90:10) + collagen microsponge	Mechanical stability	–	[207]
Nerve regeneration	Aligned PLGA fibers + alginate hydrogel contained in knitted PLA sheath coated with electrospun PLA nanofibers	Mechanical stability; Controlled poresize adjusted to nerve regeneration; Aligned fibers for cell guidance	Neurothropic factors (NT-3 or BDNF with LIF, SMDF and MGF-1)	[63]
Dermal grafts	Warp-knitted PLGA-mesh + collagen/chitosan sponge	Mechanical stability	–	[49, 54, 159]
Tendon regeneration	Knitted silk + collagen sponge	Mechanical stability; provides space for tissue ingrowth	SDF-1 alpha	[48]
Esophagus replacement	Porous collagen + PCL knitting, tubularized by sutures	Mechanical stability	–	[60]
Hernia repair	PLGA (90:10) knit + collagen sponge	Mechanical stability	–	[208]
Small joint reconstruction	Knitted poly-L/D-lactide (P(L/D)LA) 96/4	Porous environment for cell ingrowth; Mechanical strength	Clinical study	[200–204]
Calvarial bone healing	Knitted P(L/D)LA 96/4	Mechanical stability	FGF-1	[209, 210]
Weaving				
Nerve regenerattion	Biodegradable glass fabric	Mechanical stability	–	[211]
Vascular graft	Woven tubes (luminal Ø 4 mm), with double-layered PGA (core)/PLLA (sheath) fibers + collagen microsponges	Mechanical stability; 3D porous environment	–	[147, 164]
Fascia replacement/hernia repair	Handwoven meshes from native dragline silk of Nephila spp.	Mechanical stability	–	[59]
Tendon/Ligament repair	Layered PLLA fabrics; side A: smooth surface, side B: pile-finished surface	Mechanical stability; Control of cell migration/adhesion by adjusted surface structures	–	[165]
	Woven PLA pad	Mechanical stability	–	[212]
	Woven PLA (commercially available material)	Mechanical stability; Host tissue deposition	–	[166]
Braiding				
Nerve regeneration	PGA tube + collagen sponge	Mechanical stability	Clinical studies	[179–181, 176]
	Braid of PLLA and PGA yarns (1:1) + collagen coating	Mechanical stability; PLLA for prolonged reinforcement	–	[50]
	Microbraided PLGA (10:90) tubes	Mechanical stability	–	[213]
Tendon/ligament repair	Lyophilized human fascia reinforced by braided PLLA/PGA fibers	Mechanical stability	–	[171, 172]
	PLLA braid + gelatin hydrogel + collagen membrane	Mechanical stability	bFGF	[146]
	PLLA 3D square braid	Mechanical stability; Tissue ingrowth	–	[173]
Non-woven				
Nerve regeneration	poly[(R)-3-hydroxybutyrate] (PHB) non-woven	Mechanical stability	Clinical study	[196]
	Oriented silk-fibroin filaments	Mechanical stability; Aligned fibers for cell guidance	–	[195]
Cartilage repair	PGA felt + hyaluronic acid/hyaluronan	Mechanical stability; Porous environment	Allogeneic/autologous serum	[144, 145]
Tendon/ligament repair	PGA sheet	Mechanial stability; Tissue ingrowth	–	[193]
	Chitin sheet	Mechanial stability; Tissue ingrowth	–	[194]

## Conclusion

In this review, the preconditions and possibilities of textile manufacturing methods, fiber development and functionalization for the fabrication of cell-free scaffolds for in situ TE have been summarized. Studies in this field encompass a variety of engineered scaffolds from simple grafts [193] to complex multi-material scaffolds [63]. The basic benefits of fibrous scaffold architectures, namely mechanical stability, porosity and degradability, are employed in most cases. However, fibrous engineered scaffold systems stay behind the possibilities which are principally offered by textile manufacturing techniques and their combination with other manufacturing techniques. In order to make use of the whole range of favors, there are four main issues which need to be addressed: (1) Logical combination of manufacturing techniques and materials. (2) Biomaterial fiber development. (3) Adaption of textile manufacturing techniques to the demands of scaffolds for regenerative medicine. (4) Incorporation of biological cues (e.g. stem cell homing factors).

1. A crucial premise for successful scaffold development is that the choice of material, manufacturing techniques and biological cues must be dictated by the targeted repair-tissue. The paradox of expert knowledge in specific techniques on the one hand and a broad overview about the huge variety of existing materials and techniques on the other hand may only be solved if intense interdisciplinary collaboration is consequently pursued. The combination of appropriate materials, manufacturing and functionalization techniques must be derived from the desired scaffold-properties [62].
2. Besides the well-established synthetic polymers (e.g. PLA, PGA, PLGA, PBT), recent developments in biomaterial fiber engineering enable the exploitation of the favorable material properties from materials such as collagen, chitosan, regenerated silk or recombinant proteins in fibers suitable for their processing into stable spatial scaffolds [51, 80, 116, 117]. However, intensified studies concerning fiber properties and the in vitro and in vivo behavior of those newly developed fibers have to be conducted in order to use them for regenerative medicine. Furthermore, regulatory restrictions regarding the use of novel fibrous materials in the human body must be taken into account [12].
3. The possibilities offered by textile manufacturing techniques to create structures with adjusted mechanical and porous properties may only be exploited if the manufacturing method is chosen based on the demands of the targeted tissue [30]. Manufacturing methods have to be adapted in order to allow the combination of the fibrous architecture with other materials (e.g. nano-fibers, hydrogels), thus creating structurally hierarchical “hybrid” scaffolds which match the host tissue. Also, techniques by which 3D net shaped geometries (similar to AM techniques) may be fabricated from fibers are to be further developed [47], thus allowing simple fabrication of custom-shaped and patient specific fibrous scaffolds.
4. Especially for in situ TE, the incorporation and sustained release of biological cues into scaffolds is crucial for their successful application [2, 7]. Despite this fact, the incorporation of biological cues is not looked at in most cases when fiber-based cell-free scaffolds are used for in situ TE. A functioning fiber-based release-system for the sustained delivery of biological cues could help in achieving an important goal in guiding host cells to form a well-integrated functional structure [2]. Therefore, intensified research is necessary.

Depending on the type of engineered tissue and the application, clinical studies showed that cell-free fibrous scaffolds may be superior to or as well as conventional “gold standard” treatments [181, 196, 202]. Besides the obvious advantages of in situ TE (off-the-shelve scaffold availability, less cost and time consumption) it has to be considered that in terms of tissue ingrowth, tissue formation and regained functionality of regenerated tissue, cell-free fibrous scaffold systems do not always yield better results as their cell-seeded counterparts [173]. With the availability of novel biomaterial fibers with sufficient mechanical performance for textile manufacturing techniques and the appropriate addition and sustained release of cell homing factors and growth factors, in situ TE approaches using cell-free fibrous scaffolds could be elevated to various clinical applications. To make progress towards this goal, the interdisciplinary collaboration of experts in the fields of medicine, biomaterials science and textile engineering has to be consequently pursued.
